# Peritoneal recurrence in gastric cancer following curative resection can be predicted by postoperative but not preoperative biomarkers: a single-institution study of 320 cases

**DOI:** 10.18632/oncotarget.17696

**Published:** 2017-05-08

**Authors:** Fan Wu, Chunmei Shi, Riping Wu, Zhiqing Huang, Qiang Chen

**Affiliations:** ^1^ The Union Clinical Medical College of Fujian Medical University, Fuzhou, Fujian Province, PR, China; ^2^ Fujian Medical University Union Hospital, Fuzhou, Fujian Province, PR, China; ^3^ Fujian Key Laboratory of Translational Cancer Medicine, Fuzhou, Fujian Province, PR, China; ^4^ Fujian Medical University Stem Cell Research Institute, Fuzhou, Fujian Province, PR, China

**Keywords:** peritoneal recurrence, gastric cancer, curative resection, postoperative biomarkers

## Abstract

To determine the risk factors for peritoneal recurrence in gastric cancer patients after curative resection, we included 320 patients with stage I-III primary gastric cancer between January 2008 and June 2012. Data on each patient's surgical and pathological information, preoperative and postoperative tumor markers were collected and analyzed retrospectively. The risk factors for peritoneal recurrence were investigated by univariate and multivariate analysis. In patients with peritoneal recurrence, advanced T or N stage, low differentiation, vascular/lymphatic invasion, perineural invasion, and elevated postoperative CEA/CA19-9 were more common than in patients without peritoneal recurrence. Patients with peritoneal recurrence showed a worse overall survival (OS) compared to those without peritoneal recurrence. In addition, patients with peritoneal recurrence within the first year had a worse OS compared to those with recurrence after 1 year. The univariate and multivariate analyses revealed that elevated number of metastatic lymph nodes and elevated postoperative CEA and CA19-9 were three independent risk factors for peritoneal recurrence in gastric cancer patients. For patients with N3 stage and high postoperative CEA and CA19-9, we found an initial steep slope within approximately 1 year and a subsequent gentle slope in the risk curve. Combined receiver operating characteristic curve analysis using the three independent risk factors for peritoneal recurrence yielded an area under the curve value of 0.73 with 73.7% sensitivity and 64.2% specificity. Therefore, the risk factors may be associated with peritoneal recurrence after curative resection in selected gastric cancer patients.

## INTRODUCTION

Gastric cancer is one of the most common cancers and is the second leading cause of cancer-related deaths worldwide. In China, the morbidity and mortality of the disease is more than two fold higher than the world average [1]. Surgery and adjuvant therapy including chemotherapy and radiotherapy are the mainstay of treatment. However, nearly 20% of gastric cancer patients are diagnosed with peritoneal metastasis before or after surgery, and more than 50% develop peritoneal recurrence (PR) following curative resection [2]. Moreover, PR can lead to bowel obstruction or malignant ascites, resulting in a poor prognosis and decline in quality of life. Several authors [3, 4] have reported that hyperthermic intraperitoneal chemotherapy (HIPEC) or intraperitoneal chemotherapy prevent PR and improve survival among postoperative gastric cancer patients with a high risk of PR. Therefore, it is very important to identify risk factors in order to implement prophylactic measures to prevent PR by using additional adjuvant chemotherapies such as HIPEC.

However, early detection of PR remains a challenge, and currently, reliable predictors for PR of gastric cancer are unavailable in clinical practice [5]. Conventional computed tomography (CT) and positron emission tomography (PET) cannot accurately diagnose peritoneal metastasis [6]. Peritoneal lavage cytology (PLC) had been regarded as a reliable method for predicting PR but it is often criticized for having a relatively low sensitivity for detecting free cancer cells and predicting PR [7–9]. Although the American Joint Committee on Cancer (AJCC) staging system in 2010 included peritoneal cytology in the TNM 7^th^ Edition, with a positive peritoneal cytology staged as M1 disease [10], the sensitivity of PLC is low, with an incidence of positive PLC ranging from 18% to 35% [11]. Later studies proved that quantitative RT-PCR of peritoneal washes was more effective and had higher sensitivity than conventional cytological examination as a tool for predicting PR in patients with gastric cancer [12–14]. Recently, Takeno et al. showed that a 22-gene expression profile using oligonucleotide microarrays covering 30,000 human probes gene expression profile from primary gastric cancer tissues can be useful in the prediction of PR after curative surgery [15]. However, these methods had some limitations including strict requirements of sample collection, a complicated and time-consuming operation process, high cost, and false positives.

To date, there is no consensus about which clinicopathological factors or biomarkers can predict PR after curative resection. Furthermore, only a few studies have analyzed the usefulness of postoperative biomarkers in predicting PR. The prognostic effect of clinicopathological factors and postoperative biomarkers such as CEA and CA19-9 remains unclear. In the present study, through an 8-year follow-up, we aim to evaluate the risk factors of PR in patients with gastric cancer following curative resection, and in particular, to focus on postoperative biomarkers that have not been fully investigated.

## MATERIALS AND METHODS

### Patient selection and classification

The study cohort included 320 patients with non-metastatic gastric cancer who received curative resection (N2 level) at the Fujian Medical University Union Hospital between January 2008 and June 2012. Patients with prior malignancy were excluded. Data on each patient's gender, age, surgical and pathological information (including resection type, TNM stage, tumor location, differentiation, vascular/lymphatic invasion, perineural invasion [PNI]), and preoperative and postoperative tumor markers (CEA, CA19-9) were collected retrospectively. Postoperative tumor markers were analyzed 4 weeks after surgery to prevent the adjuvant therapy from affecting the outcome.

The primary tumor and regional lymph nodes were classified histologically and staged according to the AJCC TNM staging system based on postoperative pathological reports [16]. Curative resection was defined as complete removal of the primary gastric tumor, D2 resection of regional lymph nodes, and absence of any residual macroscopic tumors. D2 resection (N2 level) was defined as the removal of nodes along the left gastric artery (station 7), common hepatic artery (station 8), celiac trunk (station 9), splenic hilum, and splenic artery (stations 10 and 11). The biomarkers included CEA and CA19-9 levels in peripheral blood samples, which were collected prior to surgery and within one month after surgery.

The primary end-points were disease-free survival (DFS) and overall survival (OS) [17, 18]. DFS was defined as the time from the date of initial surgery to the first event (relapse, metastasis, or death). OS was defined as the period from initial surgery to the time of death. PR was diagnosed when disseminated nodules were found in the peritoneal cavity by imaging studies (CT, abdominal ultrasound [US], MRI, or PET-CT), ascitic cytology, laparoscopy, or laparotomy.

### Follow-up

Patients were generally seen for follow-up within the first month of surgery and then every 3 months after surgery. For patients who discontinued follow-up at our hospital, attempts were made to obtain information *via* telephonic contact. In total, 21 patients (21/320, 6.5%) were lost to follow-up, and we censored these patients in the analysis. The latest follow-up was at the end of July 2016. The median follow-up duration was 36 months (range: 3-96 months). This retrospective study was approved by the Ethical Committees of Fujian Medical University Union Hospital.

### Statistics

The chi-square test or Fisher's exact test was used to assess the distribution of clinical characteristics between patients with PR and without PR. Survival plots were drawn using the Kaplan-Meier method. Univariate analysis of the differences in prognosis between the two groups was conducted by using the log-rank test. A Cox multivariate regression model was used to identify clinicopathological factors affecting prognosis in the entire cohort. The major covariates were age, gender, type of surgery, histologic type, tumor invasion, lymph node status, tumor location, differentiation, vascular/lymphatic invasion, PNI, adjuvant therapy, and preoperative and postoperative biomarkers. Only patients with information for all of the covariates were included in the Cox multivariate regression analyses. Results that had *p*-values < 0.05 were considered statistically significant. SPSS 19.0 software was used for statistical analysis.

## RESULTS

### Clinicopathological characteristics of patients

During the follow-up period, 188 patients (188/320, 58.8%) developed metastasis after curative resection. A total of 77 patients (77/188, 41.0%) developed peritoneal recurrence; other metastatic sites included abdominal wall metastasis in 1 case, lung metastasis in 1 case, pancreas metastasis in 2 cases, bone metastasis in 5 cases, liver metastasis in 10 cases, locoregional (including anastomosis or stump, adjacent organ and regional lymph nodes) recurrence in 15 cases, distant lymph nodes (including retroperitoneal lymph nodes and extra-abdominal nodes) in 24 cases, and various combinations of multiple sites.

The clinical characteristics of the 320 patients are shown in Table 1. Gastric cancer with PR was associated with deeper depth of invasion (T stage), more number of metastatic lymph nodes (N stage), and advanced TNM stage, and the PR group had more low differentiation, vascular/lymphatic invasion, and PNI. In addition, levels of postoperative biomarkers (elevated CEA and CA19-9) were significantly positively associated with PR in gastric cancer (postoperative CEA, *p* = 0.005; postoperative CA19-9, *p* = 0.001). However, age, gender, type of resection, and tumor location were not associated with PM. In addition, elevated pre-operative biomarkers were not associated with peritoneal recurrence.

**Table 1:** Clinical characteristics of gastric cancer patients with and without PR

characteristics	Patients with PR *N* = 77	Patients without PR *N* = 243	*P*
	*n* (%)	*n* (%)	
Age (years)			0.62
<60	36	105	
≥ 60	41	138	
Gender			0.88
male	57	183	
female	20	60	
Type of resection			0.27
LTG	56	159	
LDG	21	84	
T stage			<0.001
T1/2	2	53	
T3	26	82	
T4	49	108	
N stage			<0.001
N0	5	55	
N1	6	31	
N2	13	61	
N3	53	96	
TNM stage			<0.001
I	1	33	
II	9	50	
III	67	160	
Tumor location			0.07
Proximal	18	84	
Others	59	159	
Differentiation			0.007
High/moderate	14	84	
Low	63	159	
Vascular/lymphatic invasion			0.008
Negative	47	187	
Positive	30	56	
Perineural invasion			0.006
Negative	59	218	
Positive	18	25	
Preoperative CEA			0.806 0.891 (high vs. normal)
Normal	49	160	
High	22	69	
Unknown	6	14	
Preoperative CA19-9			0.281 0.180 (high vs. normal)
Normal	53	189	
High	19	43	
Unknown	5	11	
Postoperative CEA			0.019 0.005 (high vs. normal)
Normal	50	194	
High	21	34	
Unknown	6	15	
Postoperative CA19-9			0.004 0.001 (high vs. normal)
Normal	49	190	
High	24	35	
Unknown	4	18	
adjuvant therapy			
yes	66	193	0.248
no	11	50	

LTG: laparoscopic total gastrectomy, LDG: laparoscopic distal gastrectomy

**Figure 1:**
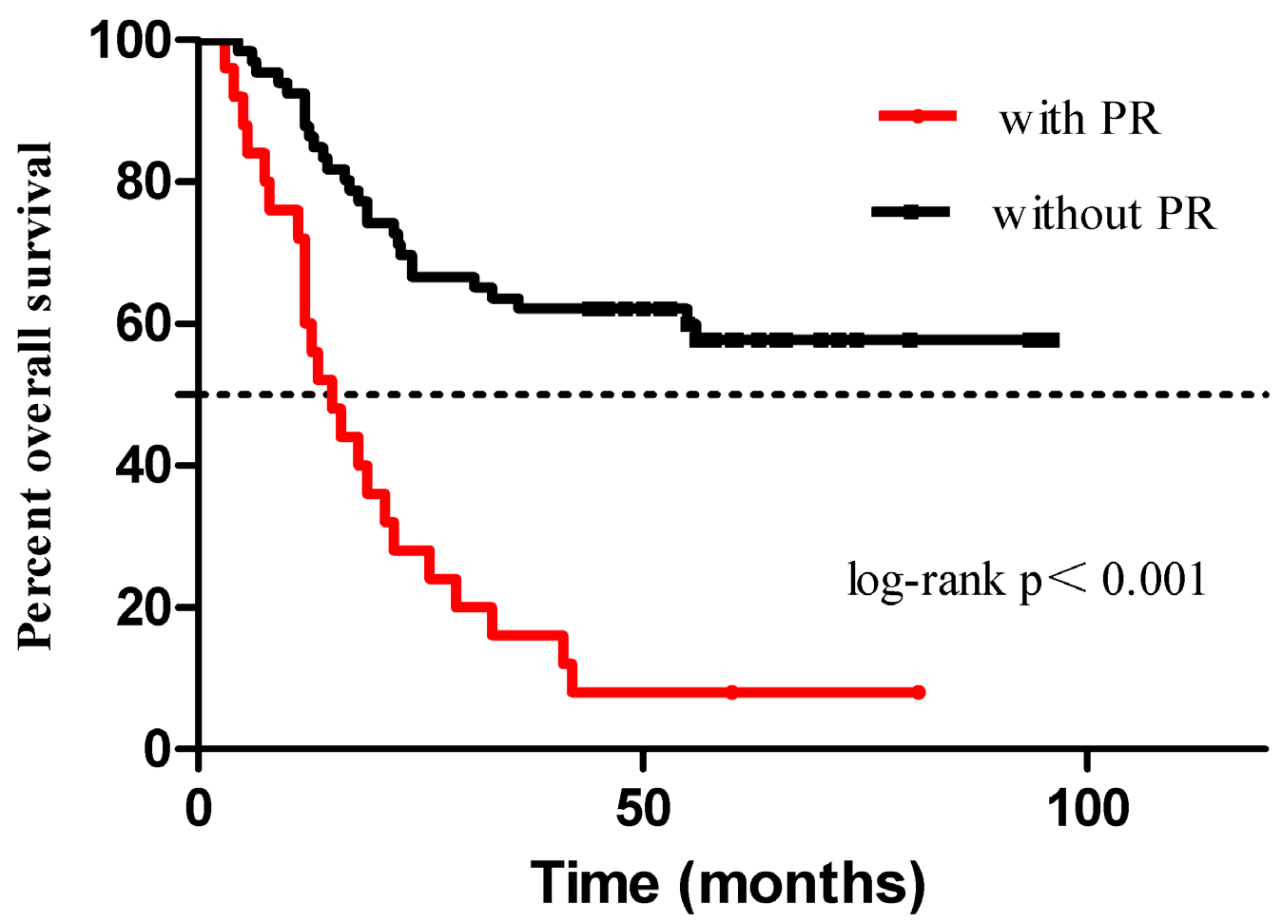
OS of patients with and without PR

In the univariate analysis, patients with PR showed a worse OS compared to those without PR, with a median OS of 15 months (95% confidence interval [CI] 9.7-20.3) *vs*. an unreached median OS (log-rank *p* < 0.001), and a hazard ratio [HR] of 7.1 (95% CI 3.4-14.8) across the entire cohort. At 1 and 3 years after surgery, patients without PR had an OS rate of 86.4% (95% CI: 78.2-94.6) and 62.1% (95% CI: 50.3-73.9), whereas patients with PR had an OS rate of 60% (95% CI: 40.8-79.2) and 16% (95% CI: 1.7-30.3), respectively. Survival plots of the two groups are shown in Figure 1.

**Figure 2:**
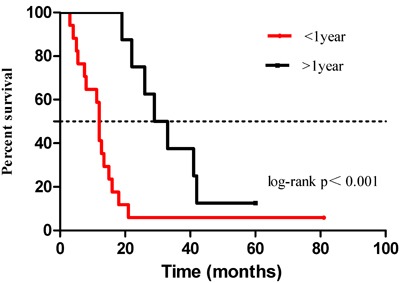
OS of patients with early recurrence and late recurrence The median overall survival time of patients with early recurrence (within 1 year) was significantly shorter than that of patients with late recurrence (after 1 year) (12 *vs*. 31 months, respectively, *p* < 0.001) for gastric cancer patients with PR.

The median time for PR was 13.3 months. Thirty-seven (37/77, 48.0%) patients developed PR within the first year after surgery, and 26 (26/77, 33.8%) patients developed PR within 1-2 years after surgery. The remaining patients (14/77, 18.2%) developed PR after 2 years following surgery (*p* < 0.001). Patients who relapsed within the first year had a worse OS than those who developed recurrence beyond 1 year, with a median OS of 12 months *vs*. 31 months, respectively, (*p* < 0.001) and an HR of 3.89 ( 95% CI 1.57-9.60). The survival plots of the two groups are shown in Figure 2.

To investigate the prognostic factors of patients with gastric cancer developing PR, we studied many potential clinicopathological factors in univariate and multivariate analysis with a Cox regression model (Table 2). The univariate analyses showed that advanced T stage, advanced N stage, poor differentiation, positive vascular/lymphatic invasion, positive PNI, elevated preoperative CA19-9, and CEA were associated with developing PR in gastric cancer patients after curative resection. The prognostic indicators identified in the univariate analyses were further studied in multivariate analyses. Cox regression analysis indicated that advanced N stage and elevated postoperative CEA and CA19-9 were three independent risk factors for PR. Furthermore, patients with 1-6 metastatic lymph nodes did not have a higher risk of PR than patients with no lymph node metastasis (*p* = 0.239), but patients with more than 7 metastatic lymph nodes showed a higher risk of PR than patients with no lymph node metastasis, with a HR of 9.43 (95% CI 2.24-39.70) (*p* = 0.002). In addition, elevated postoperative CEA and CA19-9 were prognostic factors for PR with HRs of 2.93 (95% CI 1.42-6.06) and 3.58 (95% CI 1.63-7.87), respectively. The detailed findings are presented in Table 2.

**Table 2:** Univariate and multivariate analysis of risk factors for PR

Factors	Univariate analysis			Multivariate analysis*		
	HR	95% CI	*p*	HR	95% CI	*p*
Age (< 60 *vs*. ≥ 60)	1.01	0.99-1.03	0.37	-	-	-
Gender (Male *vs*. Female)	1.00	0.60-1.66	0.987	-	-	-
Type of resection (LTG *vs*. LDG)	0.68	0.41-1.13	0.138	-	-	-
T stage (T3/4 *vs*. T1/2)	2.40	1.50-3.82	<0.001	2.60	0.26-25.78	0.414
N stage (N3 *vs*. N1/2 *vs*. N0)			<0.001			<0.001
N1/2 *v*s. N0	2.75	1.09-6.89	0.031	2.26	0.25-20.47	0.467
N3 *vs*. N0	8.68	3.69-20.41	<0.001	11.46	1.10-119.90	0.042
TNM stage (I *vs*. II *vs*. III )			<0.001	-	-	-
II *vs*.I	4.28	1.23-14.88	0.022	-	-	-
III *vs*.II	3.20	2.26-4.52	<0.001	-	-	-
III *vs*.I	3.85	2.64-5.60	<0.001	-	-	-
Location (Proximal *vs*. distal)	1.20	0.71-2.04	0.491	-	-	-
Differentiation (poor *vs*. well)	2.96	1.59-5.50	0.001	1.02	0.46-2.28	0.961
Vascular/lymphatic invasion (+ *vs*. -)	2.23	1.41-3.54	0.001	1.26	0.63-2.44	0.544
Perineural invasion (+ *vs*. -)	1.72	1.00-2.95	0.049	1.43	0.78-2.63	0.248
Preoperative CEA (high *vs*. normal)	1.24	0.68-2.26	0.49	-	-	-
Preoperative CA199 (high *vs*. normal)	1.87	1.01-3.45	0.045	1.14	0.58-2.34	0.697
Postoperative CEA (high *vs*. normal)	3.62	1.79-7.34	<0.001	3.13	1.54-6.34	0.002
Postoperative CA199 (high *vs*. normal)	4.73	2.20-10.20	<0.001	3.97	1.78-8.84	0.001

* The multivariate analysis was stratified by TNM stage because there was a positive association between high preoperative/postoperative biomarkers ( CEA and CA19-9 ) and tumor stage with low strengths (data not shown)

To further evaluate the priority of these three risk factors for predicting PR, analysis was performed in recurrent patients but not whole patient group. And we found that elevated number of metastatic lymph nodes and elevated postoperative CEA/CA19-9 were still three independent risk factors for PR within recurrent patients (Table 3)

**Table 3:** Univariate and multivariate analysis of risk factors for PR in recurrent patients

Factors	Univariate analysis			Multivariate analysis*		
	HR	95% CI	*p*	HR	95% CI	*p*
Age (< 60 *vs*. ≥ 60)	1.13	0.72-1.80	0.593	-	-	-
Gender (Male *vs*. Female)	0.82	0.48-1.40	0.427	-	-	-
Type of resection (LTG *vs*. LDG)	0.68	0.41-1.13	0.095	-	-	-
T stage (T4 *vs*. T2/3)	1.04	0.65-1.64	0.881	1.39	0.72-2.68	0.332
N stage (N3 *vs*. N1/2 )	1.68	1.07-2.61	0.029	2.39	1.10-5.17	0.027
TNM stage (I *vs*. II *vs*. III )			0.887	-	-	-
II *vs*. I	1.18	0.17-8.19	0.868	-	-	-
III *vs*.II	1.19	0.61-2.27	0.636	-	-	-
III *vs*. I	1.14	0.18-7.27	0.898	-	-	-
Location (Proximal *vs*. distal)	1.25	0.76-2.07	0.391	-	-	-
Differentiation (poor *vs*. well)	1.51	0.89-2.56	0.125	1.20	0.55-2.60	0.653
Vascular/lymphatic invasion (+ *vs*. -)	1.81	1.09-3.02	0.008	1.71	0.93-3.15	0.086
Perineural invasion (+ *vs*. -)	1.20	0.69-2.09	0.497	-	-	-
Preoperative CEA (high *vs*. normal)	1.23	0.71-2.14	0.425	-	-	-
Preoperative CA199 (high *vs*. normal)	1.63	1.01-3.43	0.011	1.14	0.43-1.78	0.703
Postoperative CEA (high *vs*. normal)	2.55	1.11-5.85	0.027	2.49	1.21-5.10	0.013
Postoperative CA199 (high *vs*. normal)	3.53	1.08-11.60	<0.001	3.45	1.48-8.08	0.004

* The multivariate analysis was stratified by TNM stage because there was a positive association between high preoperative/postoperative biomarkers ( CEA and CA19-9 ) and tumor stage with low strengths (data not shown)

**Figure 3:**
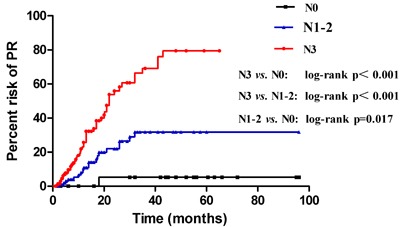
Risk of PR between three subgroups: N0, N1-2, and N3

Kaplan-Meier survival curves also revealed that an increased number of metastatic lymph node was associated with an increased rate of PR (*p* < 0.001) (Figure 3). Patients with stage N0, N1-2, and N3 had a 1-year and 2-year risk of PR of 0%, 10.85%, and 25.69% (*p* < 0.001) and 5.26%, 22.03%, and 47.46% (*p* < 0.001), respectively. Patients with N3 stage had a higher recurrence risk compared to those with N1-2 (*p* < 0.001) and N0 (*p* < 0.001) stages, respectively, and patients with N1-2 stage also had a higher recurrence risk compared to those with N0 (*p* = 0.017).

**Figure 4:**
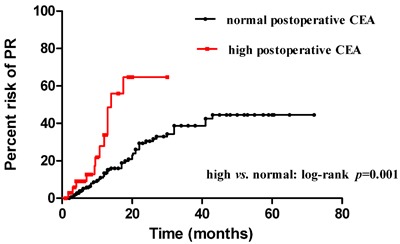
Risk of PR between two subgroups: elevated and normal postoperative CEA

**Figure 5:**
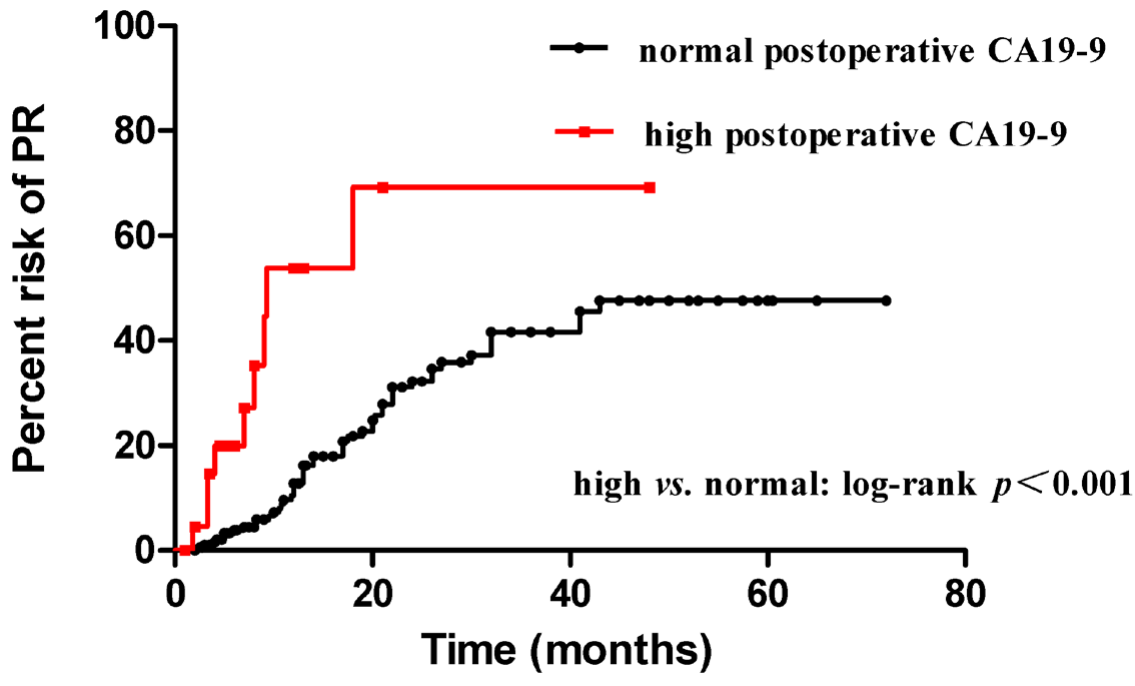
Risk of PR between two subgroups: elevated and normal postoperative CA19-9

Elevated postoperative CEA and CA19-9 were associated with a higher risk of PR compared to normal postoperative CEA (*p* < 0.001) (Figure 4). and CA19-9 (*p* < 0.001) (Figure 5). In addition, normal postoperative CEA had a 1-year and 2-year risk of PR of 13.37%, 30.46%, whereas elevated postoperative CEA had a 1-year and 2-year risk of PR of 33.88% (*p* < 0.001) and 64.74% (*p* < 0.001), respectively. Normal postoperative CA19-9 had a 1-year and 2-year risk of PR of 12.81%, 32.25%, whereas high postoperative CA19-9 had a 1-year and 2-year risk of PR of 53.78% (*p* < 0.001) and 69.19% (*p* < 0.001), respectively. For patients with N3 stage and high postoperative CEA and CA19-9, we found an initial steep slope within approximately 1 year and a subsequent gentle slope in the risk curve.

**Figure 6:**
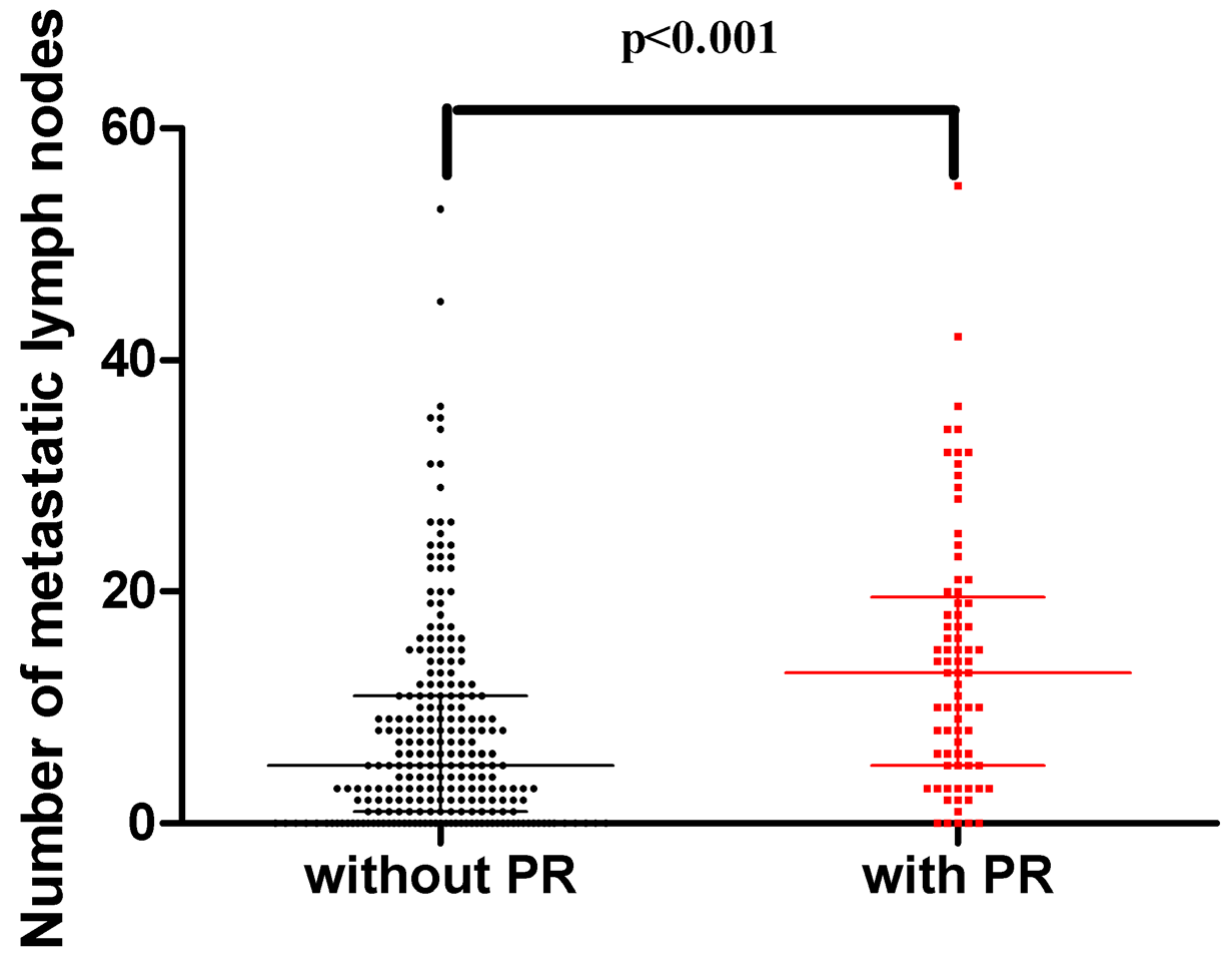
Number of metastatic lymph nodes between patients with and without PR

The number of metastatic lymph nodes between patients with and without PR evaluated by a scatter diagram(Figure 6). PR patients had a median metastatic lymph node number of 13 compared to 5 in patients without PR (*p* < 0.001)

**Figure 7:**
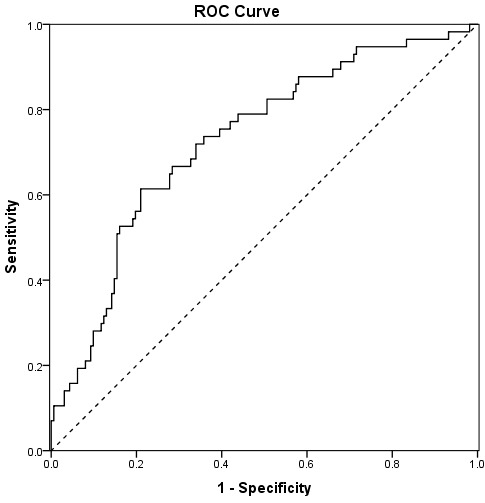
Combined ROC curve analysis using the three independent risk factors for predicting PR

Combined receiver operating characteristic (ROC) curve analysis using the three independent risk factors, including number of metastatic lymph nodes, postoperative CEA, and postoperative CA19-9 levels for predicting PR in gastric cancer patients yielded an area under the curve (AUC) value of 0.73 (95% CI 0.65-0.81) with 73.7% sensitivity and 64.2% specificity (Figure 7).

## DISCUSSION

The development of PR in patients with gastric cancer is associated with a poor prognosis, and adversely affects the quality of life [19]. Randomized trials of adjuvant intraperitoneal chemotherapy revealed a significant reduction of PR in gastric cancer patients [3, 20]. However, there are some side effects such as chemical peritonitis, pain, intestinal adhesion, etc. Therefore, it is important to find risk factors related to PR and determine a suitable population for intraperitoneal perfusion chemotherapy in patients with stage I-III gastric cancer after curative resection.

At present, it remains unclear which clinical factors can predict PR in gastric cancer patients after surgery. US, CT and PET/CT are commonly used for detecting PR [21–23]. However, a meta-analysis revealed that US and CT did not have a consistently high sensitivity and specificity in assessing PR in gastric cancer patients [24]. In addition, the 18F-FDG method for detecting PR is too expensive, although it has high sensitivity and specificity. [25]. Recent developments in staging laparoscopy have helped overcome these problems and has become the main tool to predict PR in gastric cancer patients [26, 27]. However, there is no clear indication for the use of staging laparoscopy to detect PR in gastric cancer patients, and it is an invasive procedure that cannot be routinely performed during follow-up. Therefore, by analyzing long-term outcomes, our study focused on the prognostic factors that could predict PR after curative resection in stage I-III gastric cancer patients.

In the present study, we found that PR was the most frequent pattern of metastasis after curative resection, with a frequency of occurrence of approximately 40%, which is consistent with previous studies [28–30]. Previous studies had shown that advanced TNM stage, differentiation, and venous/lymphatic invasion were more frequent in patients with PR [31, 32]. We also found that PR was associated with advanced T stage, advanced N stage, low differentiation grade of the primary tumor, and vascular/lymphatic invasion. In addition, a previous study showed that PNI positivity in gastric cancer patients after curative resection was associated with poor prognosis [33]. Furthermore, our data revealed that PNI positivity was found more frequently in patients with PR than those without PR, which was reported in only in a few previous studies. In addition, higher levels of postoperative biomarkers (CEA and CA19-9) were significantly and positively associated with PR in gastric cancer patients (postoperative CEA: *p* = 0.001; postoperative CA19-9: *p* = 0.007). These findings are consistent with those of other studies [31, 34]. However, one study did not find any such correlation between CA 19-9 and CEA, and PR [35]; these differences might be due to the D1 lymph node dissection carried out in that study, and its relatively small sample size.

Our results showed that patients with PR had a worse OS (median OS: 15 months) than those without PR (*vs* unreached median OS) using univariate analysis (log-rank *p* < 0.001; HR, 7.1; 95% CI, 3.4-14.8). Fukuchi et al. also found that patients with PR had a median OS between 14 and 17 months [36]. Results from the EVOCAPE 1, a multicentric prospective study [37], revealed that gastric cancer patients with PR had a median OS of only 6.5 months. However, more than half (73/125, 58.4%) of these patients had peritoneal carcinomatosis at the time of primary gastric cancer diagnosis, which decreased the survival rates. In addition, advances in adjuvant chemotherapy and chemohyperthermic peritoneal perfusion also improved the OS of patients with gastric cancer.

In the present study, the median time for PR was 13.3 months, which was also found in another study [38]. Our data also suggested that more than 80% of patients developed PR within 2 years of curative resection, especially within 1 year with a rate of 48.0%. Furthermore, patients who developed PR within 1 year had a worse prognosis than those who developed PR after 1 year, with a HR of 3.89 (95% CI, 1.57-9.60). Therefore, this finding indicated that more attention should be focused on PR within the first year after surgery during follow-up for those patients with high risk of PR.

To determine the independent risk factors for predicting PR in gastric cancer patients following curative resection, we performed univariate and multivariate analyses while adjusting for multiple factors. The univariate analyses showed that deeper depth of tumor invasion (T stage), more number of metastatic lymph nodes (N stage), poor differentiation, positive vascular/lymphatic invasion, positive PNI, elevated preoperative CA19-9, and elevated postoperative CEA and CA19-9 predicted PR in gastric cancer patients after curative resection. Multivariate analyses by Cox regression indicated that N3 stage and elevated postoperative CEA and CA19-9 were the three independent risk factors for predicting PR.

There have been several studies investigating the risk factors for predicting PR in gastric cancer patients, but with varied results [36, 39, 40]. Most studies found advanced T and N stage, and poor differentiation were risk factors for predicting PR; these results were also found in the present study. However, the role of serum biomarkers, like CEA or CA19-9, in predicting PR in gastric cancer patients was much more controversial. Masaki Ohi et al. indicated that preoperative CA19-9 was an independent risk factor for predicting PR [31], but other studies [35, 41] have shown different results. Studies of postoperative biomarkers [42, 43] have shown that postoperative normalized CEA or CA19-9 levels can be used as good prognostic factors in patients who undergo curative gastric resection.

However, there is no evidence on the use of postoperative serum biomarkers as predictors of PR in gastric cancer patients. Our data showed that postoperative but not preoperative biomarkers could predict PR in gastric cancer patients after curative resection. Therefore, the patients with N3 stage (more than 7 metastatic lymph nodes), elevated postoperative CEA and CA19-9 as a high-risk group for PR; following further randomized prospective studies, patients with these risk factors should be considered for receiving adjuvant HIPEC treatment.

Other studies have indicated that intraoperative lavage cytology could predict PR, but this remains controversial due to its low sensitivity [7, 44, 45]. In addition, evaluating the CEA level in peritoneal lavage fluid (pCEA) [46] or evaluating pCEA expression by RT-PCR [12, 13] were considered to be more suitable than intraoperative lavage cytology in predicting PR. However, these results also remain controversial [47]; additionally, obtaining peritoneal wash fluid is time consuming and inconvenient. Therefore, further research is needed to find predictors of PR by combining risk factors including postoperative serum biomarkers, pCEA expression by RT-PCR and other clinical pathological factors. We believe this approach would be more suitable for predicting PR [48].

To further elucidate the role of postoperative CEA, CA19-9, and metastatic lymph nodes in predicting PR, we used the Kaplan-Meier method to compare the risk of PR between different numbers of metastatic lymph nodes. Our results showed that advanced N stage was positively associated with an increased risk of PR (*p* < 0.001). Univariate analysis showed that patients with more than 7 metastatic lymph nodes (N3) had a higher risk of PR than those with N1-2 (*p* < 0.001) or N0 (*p* < 0.001); patients with N1-2 also had a higher recurrence risk compared to those with N0 (*p* = 0.017). These results also proved that N3 stage was an independent predictor of PR in patients with gastric cancer, which was also revealed by multivariate analysis. We further compared the number of metastatic lymph nodes between patients with and without PR by a scatter diagram. PR patients had a median metastatic lymph nodes number of 13 compared to 5 in patients without PR (*p* < 0.001), which suggested that the number of metastatic lymph nodes could predict PR, especially in patients with N3 stage. We also compared the risk of PR between elevated postoperative CEA and CA19-9 and normal postoperative CEA and CA19-9 by the Kaplan-Meier method. Elevated postoperative CEA and CA19-9 had a higher risk of PR compared to normal postoperative CEA and CA199, with an initial steep slope within the first year and a subsequent gentle slope in the risk curve. The results were consistent with those of other studies [42, 43]. An advantage of our study is the time of evaluation of postoperative biomarkers, which avoided the bias of adjuvant chemotherapy or chemohyperthermic peritoneal perfusion.

Next, we performed ROC curve analysis using independent clinical predictors including the number of metastatic lymph nodes, elevated postoperative CEA, and elevated postoperative CA19-9 for predicting PR in gastric cancer patients. This yielded an AUC value of 0.73 (95% CI, 0.65-0.81) with 73.7% sensitivity and 64.2% specificity for predicting PR. These findings suggested that these three independent factors could predict PR after curative resection. Although future studies with a larger cohort and longer follow-up are needed, our results might be useful in counseling gastric patients after curative resection about treatment options including adjuvant chemotherapy or chemohyperthermic peritoneal perfusion.

There are several limitations to this study. First, it is a retrospective study and must be interpreted with caution because of the increased likelihood of false-positives and false-negatives. Second, the small sample size of this study, which widens the CIs of the outcomes, is not enough to obtain a convincing result. Last, the information of PLC and the CEA level in peritoneal lavage fluid (pCEA) or pCEA expression by RT-PCR were not evaluated in the present study. Further study would be able to produce more convincing results and have greater implications for clinical practice when combined with information of PLC or pCEA.

In summary, this study suggests that patients with PR had a worse OS when compared to patients without PR, and patients who developed PR within the first year had a worse prognosis than those who relapsed after 1 year. Furthermore, elevated postoperative but not preoperative CEA and CA19-9 could predict peritoneal recurrence, and N3 stage was also an important independent predictor for PR in gastric cancer patients following curative resection. And we found an initial steep slope within approximately 1 year and a subsequent gentle slope in the risk curve for those patients with N3 stage and high postoperative CEA and CA19-9. Combined ROC curve analysis using these three independent clinical predictors could predict PR in gastric cancer patients. These findings might be helpful in selecting patients who would be eligible to receive adjuvant HIPEC. Randomized prospective studies are needed to further validate these clinical predictors.
